# The willingness of patients to make the first visit to primary care institutions and its influencing factors in Beijing medical alliances: a comparative study of Beijing’s medical resource-rich and scarce regions

**DOI:** 10.1186/s12913-019-4184-0

**Published:** 2019-06-07

**Authors:** Haiyan Song, Xu Zuo, Chengsen Cui, Kai Meng

**Affiliations:** Department of Social Medicine and Health Management, School of Public Health, Capital Medical University, Beijing, 100069 China

**Keywords:** Medical alliances, First visit to primary care institutions, Willingness

## Abstract

**Background:**

To improve the efficiency of the use of medical resources, China has implemented medical alliances (MAs) to implement a hierarchical diagnosis and treatment system. The willingness to undertake a first visit to primary care institutions (PCIs) is an important indicator of the effect of this system. Beijing has also built MAs since 2013, but to date, there have been few studies on the first visit to PCIs in Beijing. The purpose of this study is to analyze patients’ willingness to make their first visit to PCIs and its influencing factors to provide references for the realization of a hierarchical diagnosis and treatment system.

**Methods:**

Two relatively different districts with large differences in resources in Beijing, D and F, were selected, and a self-reported questionnaire and convenience sampling method were applied. A cross-sectional survey was administered to 1221 patients of MAs. The chi-square test and binary logistic regression were used to analyze the influencing factors of patients’ willingness to undertake a first visit to a PCI.

**Results:**

Fewer patients in District D received medical alliance services (44.42%) than those in District F (59.25%), but patients in District D had a higher degree of satisfaction with the services they received (72.04%) than those in District F (28.96%). Patients in District D had a higher willingness to undertake a first visit (64.00%) than those in District F (58.18%). Patients of an older age, low medical expenses, participation in urban employees’ basic medical insurance, a high understanding of MAs and high satisfaction with medical services were indicators of being more willing to choose primary care institutions for their first visit.

**Conclusions:**

The different medical resources and MA constructions in the two districts have resulted in a difference between the two districts in terms of the willingness of individuals to make their first visit to PCIs. Strengthening the service capabilities of PCIs remains a priority. The government should propose solutions to solve the problems encountered in practice and actively promote the realization of MAs and hierarchical diagnosis and treatment.

**Electronic supplementary material:**

The online version of this article (10.1186/s12913-019-4184-0) contains supplementary material, which is available to authorized users.

## Background

The purpose of medical alliances (MAs) is to promote the first visit to primary care institutions (PCIs) in China, while the effect of this reform is not satisfactory in practice. What are the factors affecting patients’ willingness to make their first visit to PCIs in Beijing? Chinese medical institutions mainly include hospitals and PCIs. Hospitals are divided into three levels according to the number of beds and functions. Class-one hospitals provide medical care directly to communities with a certain population and have less than 100 beds. Class-two hospitals provide comprehensive medical services and have between 101 and 500 beds. Class-three hospitals provide high-level specialist medical services to several regions and perform higher education and scientific research tasks, these hospitals have over 500 beds. PCIs are community health service centers (stations) in cities, township health centers and village clinics in rural areas [1]. However, the distribution and utilization of health resources in China is unreasonable [2]. There are significant differences in medical resources between urban and rural areas, and most health resources are concentrated in Class-three hospitals, while PCIs lack high-quality medical resources [3]. In addition, due to the high demands for health and the lack of trust in PCIs [4], patients definitely prefer Class-three hospitals to PCIs [5]. The current disordered situation in medical treatment in China has led to two results. First, high-quality medical resources in Class-three hospitals are mostly used to treat common and frequently occurring diseases [6], while these hospitals’ teaching and scientific research functions are hampered by a busy clinical practice [7]. Furthermore, PCIs are left idle on a large scale, and medical resources are wasted, which makes it difficult and expensive for people to seek medical treatment.

By the end of 2016, there were 0.93 million PCIs in China, 0.62% higher than in 2015 but far lower than the 6.82% growth in the number of hospitals [8]. In 2016, outpatient and emergency visits in PCIs accounted for only 56.14% of the total outpatient and emergency visits, while the proportion of hospital visits remained high, and 20.6% of the patients were still concentrated in 0.2% of all Class-three hospitals [9]. This reluctance to visit PCIs leads to a continuously increasing volume of hospital medical services and will severely affect the implementation of a first visit to PCIs [10].

To improve the utilization efficiency of medical resources and maximize their effectiveness, China has promulgated a series of policies to promote a reform of the medical system and has gradually established a hierarchical diagnosis and treatment system to meet people’s health needs. The system classifies diseases according to their severity and urgency and enables different levels of medical institutions to handle different types and stages of diseases according to the institution’s function. The order of medical treatments has improved through first visits to PCIs and two-way referral systems, ultimately achieving the maximum utilization of medical resources [11].

To establish a hierarchical diagnosis and treatment system, China has also implemented regional medical alliances (MAs) [12], medical groups [13], hospital trusteeships [14], and hospital-establishing and hospital-managing modes [15]. A MA is a vertically coordinated service organization comprising high-level medical institutions, including Class-three and Class-two hospitals within a certain area and PCIs, such as community health service organizations, township hospitals and village clinics [16]. Although the emergence of a variety of MAs has partially solved the problem of the allocation of medical resources in PCIs and has promoted the implementation of two-way referrals, various problems remain. Patients’ willingness to make their first visit to PCIs is still low [17].

As the capital of the country, Beijing is rich in high-quality medical resources, which leads to a very large number of patients in Class-three hospitals. In 2008, the number of patients treated at tertiary hospitals in Beijing was 41.4 million, reaching 80.7 million by the end of 2012, representing an increase of 94.83% [18]. To solve this problem, Beijing issued the Guidelines for the Pilot Project for the Construction of the Beijing Regional MAs [19] in November 2013. By the end of January 2018, 58 MAs had been established in Beijing. However, because the distribution of medical resources among different regions and among medical institutions at all levels is uneven, local patients in Beijing tend to receive treatment in Class-three hospitals. On the other hand, the medical insurance of residents in China mainly includes urban employees’ basic medical insurance (UEBMI), urban residents’ basic medical insurance (URBMI), new rural cooperative medical system (NCMS) and commercial insurance (CI), with the first three medical insurances covering the entire population. There is little difference in the proportion of reimbursement between URBMI and NCMS when patients visit large hospitals or PCIs [20]. Therefore, patients are still more willing to visit a large hospital with high medical standards. In 2016, the number of visits to PCIs in Beijing was 56 million, which is still far below the number of outpatient and emergency services in hospitals: 145 million [21]. The rate of patients’ first visits to PCIs is too low to achieve the goal of hierarchical diagnosis and treatment.

The low willingness to make a first visit to PCIs is the main reason preventing the realization of hierarchical diagnosis and treatment. A literature review found that in a survey of 6 cities in China in 2013, 62.2% of patients felt uncomfortable choosing PCIs for their first visit [22]. A survey in Zhuhai City in 2016 showed that residents’ willingness to attend PCIs for their first visit was 76.43% [23]. In a survey of residents’ intentions in Xuzhou in 2017, 58.50% of residents were willing to attend PCIs [24]. However, there has not yet been a study performed on Beijing residents regarding their willingness to make their first visit to PCIs. On the other hand, the authors believe that the uneven distribution of medical resources might be an important factor affecting the construction of MAs and that the willingness to make a first visit to PCIs in regions with good medical resources might be higher. Therefore, this study selects the two urban districts with the largest differences in the distribution of medical resources for analysis. From the patients’ perspective, this study analyzed the differences in patients’ willingness to make their first visit to PCIs in the two districts and its influencing factors and explored the factors constraining the achievement of hierarchical diagnosis and treatment. Finally, this study provided some suggestions as to how to establish MAs to develop a hierarchical diagnosis and treatment system in Beijing and even China.

## Methods

### Study design and study areas

A cross-sectional study based on an organized survey was conducted in two districts of Beijing. There are 16 districts in Beijing, and the medical resources of each district are very different. Two districts, D and F, with a large gap in medical resources, were selected for analysis. District D has a permanent population of 0.90 million and an area of 41.86 km^2^. District D is rich in quality medical resources: there are 9 tertiary hospitals, 1 tertiary integrated Chinese and Western medicine hospital, 8 secondary hospitals, 37 Class-one hospitals, 7 community health service centers and 58 community health service stations. As the lead hospitals, the four tertiary hospitals in the district have established 4 MAs to integrate the medical resources of the public hospitals in the whole district. District F has a permanent population of 2.32 million and an area of 305.80 km^2^. There are 4 Class-three hospitals, 7 Class-two hospitals, 45 Class-one hospitals, and 18 community health service centers in District F. The medical resources are relatively scarce, especially comprehensive tertiary hospitals. Therefore, District F established five regional MAs based on the distribution of residents and the distribution of medical institutions, and the 5 Class-two hospitals in this district are used as core hospitals, which form a connecting link between the 6 top Class-three hospitals (located in other districts of Beijing) and the 15 community health service centers (Table 1).

**Table 1:** The distribution of health resources in Beijing in 2015

Variables	District D	District F
Population size (ten thousand people)	90.50	232.40
Occupied area(km^2^)	41.86	305.80
Municipal GDP per capita(ten thousands RMB)	23.48	5.75
Practicing (assistant) physician per thousand people	10.82	2.80
CHS professionals per thousand people	12.78	10.22
General practitioner per thousand people	5.58	4.49
Number of hospitals	65	70
Number of Class-three hospital	9	4
Number of Class-two hospital	8	7
Number of Class-one hospital	37	45
Number of Community health service centers/station	65	173

### Study population and sample

All the MAs in these two districts were selected to conduct this research, and patients treated in community health service centers/stations in these MAs were selected as the study population. There are 7 community health service centers and 58 community health workstations in District D. A convenience sampling method was used to select 50 patients from each community health service center and 3 patients from each community health station, for a total of 524 patients in District D. Fifteen community health service centers participated in the construction of the MAs in district F, and 50 patients were selected from each community health service center, for a total of 750 patients. In total, there were 1274 subjects selected in the two districts.

### Questionnaire

To analyze the willingness of patients to make their first visit to PCIs, a questionnaire was designed based on the existing literature and on the themes of the interviews. Three personal in-depth interviews were held in the two districts, which included 7 different staff members in each interview. Through an analysis of the interview results, this study found the current status of the MAs, including the reason for patients’ visits and the medical services patients desire. Through a literature review, this study also found that patients’ understanding [25] of the policies of MAs and their satisfaction [26] with the medical services of MAs are factors that influence the willingness of patients to make their first visit to PCIs. The goal of the reform is to increase the number of patients making a first visit to PCIs [27]. Therefore, patients’ willingness to make a first visit to PCIs, their level of understanding of the policy, their satisfaction with the medical services, and their reasons for using primary health care were investigated in the questionnaire.

The complete questionnaire had 23 entries (see Additional file 1). The content of the questionnaire included the sociodemographic characteristics of the residents, their degree of understanding of the MAs’ policies, the willingness of patients to make a first visit to PCIs, and their satisfaction with and evaluation of the MAs’ services. We asked the respondents to provide their sociodemographic characteristics in detail. A 5-point Likert scale was applied to investigate the degree of understanding of the MAs, the degree of satisfaction with the MAs, and the willingness to make a first visit to PCIs. Multiple-choice questions were used to determine the reasons for the respondent’s choice of medical institution and evaluation of the MAs. A pre-survey of 50 respondents was conducted before the formal survey to test its external and internal validity. The questionnaire was designed to be completed in 10–15 min. Amendments were made based on the feedback received.

### Data collection

To ensure the quality of the survey, the questionnaires were arranged by the District Health and Family Planning Commissions. The survey was administered to patients of the community health centers/stations in the MAs, and 1274 questionnaires were distributed. In total, 475 valid questionnaires were returned from District D, 746 valid questionnaires were returned from District F, and the total valid response rate was 95.84%. EpiData3.1 software was used to double the input data and control the consistency of the input data.

## Measures

The dependent variable was the willingness of patients in the MAs to make their first visit to PCIs (That is, “Are you willing to make a first visit to PCIs?” The term “PCIs” in this article refers to the community health service centers/stations.). In the statistical analysis, this five-category variable was combined into binary variables: the two options “very willing” and “more willing” were merged into “willing”; “indifferent”, “more unwilling” and “unwilling” were combined into “unwillingness”.

The independent variables included the sociodemographic characteristics (including region, gender, age, domicile, per capita monthly medical expenditures, chronic disease condition, and types of medical insurance participation), the cognition of the MAs (the degree of understanding of the MAs and the means of obtaining understanding), and the satisfaction with the MAs.

### Statistical analysis

The data were analyzed using IBM SPSS Statistics V20. First, we analyzed the data from the two districts and performed descriptive analyses of the sociodemographic characteristics of the residents, the degree of understanding of the MAs’ policies, the willingness of the patients to make a first visit to PCIs, and the satisfaction with the MAs’ services in the two districts. The chi-square test was used to examine the differences in the distribution of these variables between the two districts. Next, a logistic regression model was generated to assess the relationship between patient’s willingness to make a first visit to PCIs and the associated factors. Step 1 (Model 1) was mainly the relationship between the area and the willingness to undertake a first visit. Step 2 (Model 2), the degree of satisfaction with the MAs’ services, was added. Step 3 (Model 3), the degree of understanding of the MAs’ policy, was added. Step 4 (Model 4), the demographic variables, was added. All of the data on the factors influencing first-visit willingness were analyzed, and *P* < 0.05 was considered statistically significant.

## Results

A total of 1221 patients participated in the study (95.84% response rate). Table 2 presents the distribution characteristics of the research variables. The majority of patients in District D were over the age of 60, while District F had more patients aged between 21 and 40. Patients whose monthly medical expenditures were below 300 Yuan comprised the largest group in district D, while in district F, the largest group had medical expenditures between 300 and 500 Yuan. The number of patients participating in basic medical insurance for urban employees in the two districts was the highest, but the number of patients with this type of medical insurance was significantly higher in District D than in District F; the number of patients participating in new rural cooperative medical systems in District F was higher than that in District D.

**Table 2:** Comparison of characteristics among patients in two districts

Characteristics	Total (*N* = 1221) N(%)	District D(*N* = 475) N(%)	District F(*N* = 746) N(%)	*P* Value χ2 test
Gender				0.97
Male	556(45.54)	216(45.47)	340(45.58)	
Female	665(54.46)	259(54.53)	406(54.42)	
Age (years)				< 0.001
0–20	4(0.33)	3(0.63)	1(0.13)	
21–40	200(16.38)	58(12.21)	142(19.03)	
41–60	578(47.34)	223(46.95)	355(47.59)	
61–80	414(33.91)	176(37.05)	238(31.9)	
81–100	25(2.05)	15(3.16)	10(1.34)	
Registered permanent residence				0.67
The city’s downtown	1075(88.04)	423(89.05)	652(87.4)	
The city’s suburbs	71(5.81)	26(5.47)	45(6.03)	
Out-of-town	75(6.14)	26(5.47)	49(6.57)	
Average monthly medical Expense (yuan)				< 0.001
< =300	408(33.42)	195(41.05)	213(28.55)	
301–500	317(25.96)	95(20)	222(29.76)	
501–800	264(21.62)	74(15.58)	190(25.47)	
801–1000	122(9.99)	50(10.53)	72(9.65)	
< 1000	110(9.01)	61(12.84)	49(6.57)	
Health insurance				< 0.001
UEBMI	639(52.33)	290(61.05)	349(46.78)	
URBMI	321(26.29)	133(28.00)	188(25.2)	
NRCMS	177(14.5)	16(3.37)	161(21.58)	
NMI	43(3.52)	24(5.05)	19(2.55)	
CI	13(1.06)	4(0.84)	9(1.21)	
Out of pocket	15(1.23)	3(0.63)	12(1.61)	
Chronic disease status [24]				0.27
No chronic disease	323(26.45)	134(28.21)	189(25.34)	
Any chronic disease	898(73.55)	341(71.79)	557(74.66)	

Note: willing/unwilling to make primary visit at the primary is a multiple choice of questions, so it is not necessary to make the χ2 test

Table 3 shows that patients in the two districts differed in the distribution of their degree of understanding and in their approaches to MAs. The degree of patients’ understanding of the MAs in both districts was low. In total, 33.05% of patients who were not familiar with the related policies of the MAs comprised the majority in District D. Patients in District F who generally understood the medical-related policies represented 50.13% of the entire group. The patients in the two districts learned the related policies of the MAs mainly from community advocacy. District F had more patients who learned the related policies through media campaigns and hospital promotion than District D. Table 4 shows that more patients had received MAs’ services in District F, while most patients in District D did not receive them. Next, we investigated and found that patients who received MAs’ services in the two districts had different levels of satisfaction. Most patients in District D indicated that they were satisfied (48.34%) or very satisfied (23.70%) with the MAs’ services, while more than half of patients in District F stated that they were generally satisfied with the services.

**Table 3:** The Cognition of medical alliances in patients in two districts

Characteristics	Total(*N* = 1221) N(%)	District D(*N* = 475) N(%)	District F(*N* = 746) N(%)	*p* Value χ2 test
Understanding level of Medical alliance				< 0.001
Do not understand very much	284(23.26)	152(32.00)	132(17.69)	
Not very understanding	261(21.38)	157(33.05)	104(13.94)	
General understanding	461(37.76)	87 (18.32)	374(50.13)	
More understanding	177(14.50)	52(10.95)	125(16.76)	
Very much understanding	38(3.11)	27 (5.68)	11(1.47)	
The ways of Understanding				< 0.001
Media reports	209(17.12)	71 (14.95)	138(18.50)	
Community promotion	444(36.36)	165(34.74)	279(37.40)	
Hospital promotion	146(11.96)	29 (6.11)	117(15.68)	
Relatives and Friends Recommend	55(4.50)	14 (2.95)	41(5.50)	
Others	83(6.80)	44 (9.26)	39(5.23)

**Table 4:** The experience and satisfaction of medical alliances in patients in two districts

	Total(*N* = 1221) N(%)	District D(*N* = 475) N(%)	District F(*N* = 746) N(%)	*p* Value χ2 test
Previous medical alliance treatment Experience				< 0.001
Yes	653(53.48)	211(44.42)	442(59.25)	
No	568(46.52)	264(55.58)	304(40.75)	
Level of satisfaction				< 0.001
Not satisfied	17(1.39)	2(0.42)	15(2.01)	
Less satisfied	49(4.01)	18(3.79)	31(4.16)	
Generally	307(25.14)	39(8.21)	268(35.92)	
More satisfactory	213(17.44)	102(21.47)	111(14.88)	
Very satisfied	67(5.49)	50(10.53)	17(2.28)	

Table 5 shows that patients in District D had a higher willingness to make their first visit to PCIs than patients in District F. The reasons for being willing to make a first visit to PCIs in the two districts mainly focused on the convenience of the PCIs and the high proportion of reimbursement. However, some patients in both districts were reluctant to make a first visit to PCIs, mainly because they claimed that it was a waste of time, while most of the patients in District F said there were fewer types of drugs available in PCIs.

**Table 5:** The willingness and reasons of first visit at primary care institutions in patients in two districts

	Total(*N* = 1221) N(%)	District D(*N* = 475)N(%)	District F(*N* = 746)N(%)	*p* Value χ2 test
The willingness of the first visit				0.04
Unwilling	483(39.56)	171(36.00)	312(41.82)	
Willing	738(60.44)	304(64.00)	434(58.18)	
Reasons for first visit at primary				
Convenience	971(79.52)	409(86.11)	562(75.34)	
The high proportion of Medicare Reimbursement	667(54.63)	246(51.79)	421(56.43)	
Suitable environment	349(28.58)	109(22.95)	240(32.17)	
Can be referred to a major hospital	248(20.31)	107(22.53)	141(18.90)	
Ensure continuity of treatment	293(24.00)	98(20.63)	195(26.14)	
Short waiting time	274(22.44)	72(15.16)	202(27.08)	
Detailed inquiry of doctors	157(12.86)	55(11.58)	102(13.67)	
Family doctor-style services	134(10.97)	52(10.95)	82(10.99)	
Prescription policy	107(8.76)	40(8.42)	67 (8.98)	
Others	4(0.33)	2(0.42)	2(0.27)	
Reasons for first visit not at primary				
A waste of time	742 (60.77)	309 (65.05)	433 (58.04)	
Distrust	649 (53.15)	275 (57.89)	374(50.13)	
Few drugs	689 (56.43)	182 (38.32)	507 (67.96)	
Lack of inspection items	473 (38.74)	141 (29.68)	332 (44.50)	
Less reimbursement	251 (20.56)	123 (25.89)	128 (17.16)	
No beds	167 (13.68)	65 (13.68)	102(13.67)	
Others	5 (0.41)	0 (0.00)	5 (0.67)	

Table 6 presents the odds ratios (ORs) among the independent variables in the willingness to make a first visit to PCIs. The regional factors were associated with statistically significant differences in patients’ willingness in Model 1. Patients in District D had a higher willingness to make a first visit to PCIs than patients in District F. The degree of satisfaction with the MAs affected patients’ first-visit willingness in both Model 3 and Model 4. The higher the degree of satisfaction with the MAs, the higher the patient willingness to make a first visit to PCIs. The degree of understanding of MAs and the ways in which patients learned about the alliances resulted in a statistically significant difference in the willingness of patients in both Model 3 and Model 4. The better the knowledge of MAs, the higher the willingness to make a first visit to PCIs. Patients who understood the MAs through community publicity, hospital promotion, and referrals of friends and relatives were more willing to make their first visit to PCIs. Model 4 shows that patients who were older, had lower expenditures, and participated in urban residents’ medical insurance and the new rural cooperative medical system were more willing to make a first visit to PCIs.

**Table 6:** The effects of societal determination on willingness of first visit at primary care institutions (OR, 95% CI; *n* = 1221)

Variables	Model 1	Model 2	Model 3	Model 4
Region				
District F	1	1	1	1
District D	1.28 (1.01–1.63)^*^	1.23 (0.96–1.56)^*^	1.29 (0.99–1.65)^*^	1.19 (0.90–1.57)^*^
Level of satisfaction		1.06 (0.97–1.15)^*^	1.03 (0.95–1.12)	1.03 (0.94–1.13)^*^
Level of understanding			1.24 (1.04–1.48)^*^	1.32 (1.09–1.59)^**^
The ways of Understanding				
Media reports			1	1
Community promotion			1.43 (1.03–2.00)^*^	1.38 (0.98–1.96)^*^
Hospital promotion			1.67 (1.01–2.61)^*^	1.67 (1.06–2.65)^*^
Relatives and Friends Recommend			2.07 (1.09–3.92)^*^	2.42 (1.23–4.74)^*^
Others			1.79 (1.05–3.08)	1.56 (0.89–2.74)
Gender				
Male				1
Female				1.03 (0.81–1.32)
Age (years)				1.54 (1.29–1.84)^**^
Registered permanent residence				
The city’s downtown				1
The city’s suburbs				1.02 (0.61–1.71)
Out-of-town				1.26 (0.73–2.18)
Average monthly medical expense (yuan)				0.88 (0.79–0.97)^*^
Health insurance				
UEBMI				1
URBMI				0.54 (0.41–0.72)^**^
NRCMS				0.61 (0.42–0.90)^*^
NMI				0.67 (0.35–1.30)
CI				0.35 (0.11–1.12)
Out of pocket				0.54 (0.18–1.67)
Chronic disease status				
No chronic disease				1
Any chronic disease				1.24 (0.90–1.71)

Note: *p* < 0.05, ^*^; *p* < 0.01, ^**^

## Discussion

Patients in the two districts showed different levels of willingness to make their first visit to PCIs. This study showed that that patients in District D had a higher willingness to visit to PCIs than patients in District F. The factors affecting patients’ willingness to make their first visit to PCIs included regional factors, satisfaction with and cognition of the MAs, age, monthly medical expenditures and type of health insurance. Therefore, we discuss the issues with the above aspects in mind.

This study showed that patients in District D had a higher willingness to attend PCIs for their first visit than patients in District F. The breadth and extent of technical support provided by core hospitals to other PCIs influenced the effectiveness of the implementation of MAs. The medical institutions in District D under the leadership of the district government closely cooperate with four powerful appointed hospitals, including Hospital X, to form regional MAs that cover the entire region. The tertiary hospital provides comprehensive business guidance and technical guidance through doctors’ visits to member organizations of the MAs for consultation and ward rounds, which improve the diagnosis and treatment capabilities and comprehensive medical service capabilities of the secondary hospitals and PCIs. The improvement of the medical level is the main factor that attracts patients to PCIs. Therefore, patients in district D were more willing to go to PCIs. Due to the lack of a strong, comprehensive tertiary hospital in District F, it has established partnerships with six top tertiary hospitals in other districts, including the F Hospital of Peking University, under the guidance of the district government. These tertiary hospitals provide technical support to district-level secondary hospitals in key disciplines or departments, and secondary hospitals provide services such as doctors’ visits and referrals to the PCIs. Because these tertiary hospitals are not located in District F, the inconvenience of transportation has led to restrictions on the effectiveness of referrals and assistance in the primary medical institutions, resulting in an insufficient capacity of PCIs; thus, only 58.18% of patients in District F are willing to make a first visit to PCIs, which is lower than the willingness of patients in District D.

Models 2 and 4 show that for patients in the two districts, the more satisfaction they have with the MAs, the more willing they are to attend a PCI for their first visit. During their operation, the MAs have taken various measures, such as enhancing the service capabilities of PCIs and smoothing the referral channels, to reduce patients’ pain, effectively resolve patients’ demands, and make patients’ MA experience more satisfactory. In this way, patients could accept their first visit to PCIs as preferable. In addition, the reasons for the unwillingness of patients in District D to make a first visit to PCIs were focused on the time wasted and distrust of PCIs, indicating that District D still needs to further strengthen its capacity for PCIs. District F patients were primarily reluctant because of the small variety of drugs and the wasted time in PCIs. This result also reflects the dissatisfaction of patients with the capabilities of PCIs. Lin et al. [28] studied the Ruijin-Luwan MAs in Shanghai and found that the low level of diagnosis and treatment in these PCIs became the main reason preventing MAs from guiding residents to seek reasonable medical treatment (53.24% of the subjects were not satisfied with their diagnosis and treatment). Due to the limited medical capabilities of PCIs, patients did not trust them and were reluctant to make their first visit to them, and patients were reluctant to be transferred to the PCIs after their disease was controlled at a hospital. This result runs counter to the hierarchical diagnosis and treatment system established by the new medical reforms [29] and attracts patients to tertiary hospitals, further exacerbating the problem of patient accumulation and the difficulty in accessing medical services at tertiary hospitals. To solve this problem, enhancing the strength of PCIs should be a priority to fulfill the residents’ health demands. Only in this way can we ensure that patients’ first visit to PCIs will not result in a waste of medical resources and in residents’ dissatisfaction and resistance. This study recommends taking measures to strengthen the ability of PCIs by introducing and cultivating high-level physicians, improving their medical service capabilities, and increasing government financial subsidies [30].

The degree of understanding of the MAs in both districts is very low. Only 16.63% of patients in District D said that they knew and understood the relevant policies of the MAs, and only 18.23% of patients in District F said that they had a better understanding of the relevant policies. It can be seen from Models 3 and 4 that the more patients know about the MAs, the more willing they were to attend PCIs for their first visit. It is suggested that we should strengthen the propaganda regarding MAs in the two districts and promote residents’ willingness to make their first visit to PCIs. This finding also shows that the three publicity methods of community propaganda, hospital propaganda, and family and friend recommendations are effective. This study shows that patients in both districts mainly learned about the relevant policies of the medical alliances through community propaganda. In District F, through the hospital’s publicity, more patients knew about the relevant MAs’ policy than patients in District D. Patients’ understanding of the relevant policies through the hospitals’ publicity of the MAs in District F was higher than that in District D. The above results might be due to patient’s trust in their doctors, and thus, the effect of the hospital propaganda would be rewarding [31]. The effectiveness of PCI publicity could also help patients understand, at the point of contact with the institution, that the diagnosis and treatment levels of the doctors in primary institutions have improved, making patients willing to go to them for their first visit. Recommendations of relatives and friends are more credible. Previous studies have shown that patients’ trust in PCIs will affect their choice of medical institutions. Therefore, the institutions should improve their service capacities to solve problems for patients and obtain a high reputation via word-of-mouth advertising.

Model 4 shows that older patients are more willing to visit primary health care institutions, possibly because older patients have a high risk of chronic diseases and require long-term medication. The statistical results regarding the age distribution of patients in the two districts also showed that both districts had the highest number of patients aged 41–60, followed by patients aged 61–80 years. This result reveals the severity of aging in Beijing. Moreover, an earlier study showed that 33% of Chinese aged 60 years and older had chronic pain [32], and thus, the health problems of older patients are also an important component of the current task in the community. At the same time, older patients may not be able to maintain their physical strength to frequently visit a large hospital. Therefore, older patients may receive long-term treatment at a CHC after receiving prescriptions from large hospitals [33]. The CHC was in close contact with the hospital in this process to obtain patients’ relevant treatment information and facilitate patient’s subsequent treatment. Young patients rarely became sick, and they therefore had less exposure to hospitals and CHC. Young patients were rarely concerned with the related medical information because of their busy work schedule. Most young patients obtained their understanding of medical alliances from media publicity, took a wait-and-see attitude toward the relevant policies and were not willing to waste their time at an institution they distrusted. Therefore, if young people became sick, they directly went to the hospital rather than the CHC. Thus, we should begin by enhancing the strength of PCIs. It is recommended that the state vigorously train general practitioners and keep good general practitioners in the community [34]. Second, the CHC should work hard to increase its ability to cure common diseases in older patients, to cooperate with core hospitals and to prepare enough medicines for the community. Doctors in core hospitals could visit the CHC on a regular basis. Effective management of older patients in the jurisdiction could not only divert patients to the community but also reduce the burden on large hospitals and gradually achieve hierarchical diagnosis and treatment. For young patients, we should first increase the media’s propaganda for the MAs and pay attention to improving publicity methods to enhance young patients’ sense of the MAs’ identity. These policies would allow these patients to correctly understand the service capabilities of primary health care institutions and would guide young patients to make a first care contact there.

The results of the study show that patients with lower medical expenses after reimbursement of medical insurance are more willing to make their first visit to PCIs. The price advantage of the large proportion of medical insurance reimbursement is the main reason for first PCI visits in both selected districts, which is in line with the convenience, economy, and efficiency of PCIs. Most of the diseases with low medical expenditures were minor illnesses, and minor illnesses should meet the requirements for hierarchical diagnosis and treatment at PCIs. A total of 41.05% of patients in District D had monthly medical expenses of less than 300 Yuan, while patients in District F were evenly distributed among the various expense ranges. The lower the medical expenditures, the higher the patient’s willingness to attend PCIs, which also supports the phenomenon that patients in District D were more likely to make a first visit to PCIs than patients in District F. Wu et al. [35] found that cost had no effect on patients’ choice of a first care institution. Residents cared more about the ability of PCIs to carry out treatment. This finding is inconsistent with those of the current paper, and the reason for this inconsistency might be the differences in the region and times of implementation. This finding also suggests that we should pay attention to improving the service capacity of PCIs while guiding patients to these institutions for medical treatment. We recommend using the medical care reimbursement quota to increase the price gap among medical institutions at all levels and guide patients to seek medical treatment according to their condition. Patients’ judgment of their condition requires the country to formulate corresponding standards for the medical institution’s diagnosis and treatment grading and to educate patients through various means to improve their health literacy [36] so that they can scientifically select a consulting institution.

This study showed that participating in different types of medical insurance affected patients’ primary care wishes to a certain extent. Patients who participated in the urban employees’ basic medical insurance were more willing to attend PCIs than those who participated in urban residents’ basic medical insurance and the new rural cooperative medical system. There was little difference in the proportion of reimbursement of URBMI and NCMS when patients went to large hospitals or PCIs. It is difficult for medical insurance policies to use price leverage to guide patients to seek medical care. Therefore, patients who participated in these two types of insurance were even less willing to make a first visit to PCIs, which increased the accumulation of patients in large hospitals and was not conducive to increasing their willingness to make a first visit to PCIs [37]. Zhang Peng et al. [38] found that providing certain medical insurance benefits for patients can best attract them to participate in the MAs (40.98%). The United States also clearly stated in the National Action Plan that health insurance should be more supportive of community-based projects to improve the accessibility and fairness of health services [39]. It is recommended that medical insurance policies and procedures be adjusted according to the current problems in the development of MAs and that necessary interest linkages should be forged so that each member unit in the MAs can establish a long-term interest cooperation mechanism. First, the medical insurance payment model should be reformed, differentiated fees and reimbursement standards for different levels of medical institutions should be implemented the gap to guide patients’ medical behavior should be gradually widened. Second, connections based on the total amount of medical insurance among the Mas should be established. We could refer to the measures adopted by the Anhui Medical Community Model [40] in terms of medical insurance to uniformly prepay the total amount of medical insurance at all levels of medical institutions (prepaid per capita) to its board of directors. We could then allocate the total amount of medical insurance in accordance with the quality of the services and satisfaction of each medical institution [41].

There are two limitations in this study that must be noted. First, this study used the convenient sampling method to administer surveys to patients, which might cause selection bias. To reduce bias, all community health service centers/stations of MAs in both districts were selected, and using large sample surveys could offset this limitation to some extent. Second, this study only investigated patients in the Beijing community. The generalizability of our results may be limited because of the particularity of Beijing as the capital of China.

## Conclusion

This study selected the two districts with the largest differences in the distribution of medical resources in Beijing and explored the differences in patients’ wishes and in the factors that influence making their first visit to PCIs with MAs in the two districts. District D, which has a wealth of medical resources, was more effective in the construction of MAs, and patients in District D are more satisfied with the MAs and more willing to make their first visit to PCIs. Older patients and patients who know more about MAs are more willing to visit PCIs. To a certain extent, the current health insurance policy affects the patients’ willingness to make a first visit to PCIs. Community propaganda, hospital promotion, and the recommendations of relatives and friends are effective means of propaganda for MAs and effectively promote a first visit to PCIs. We suggest strengthening the capacity of PCIs, improving the referral and division mechanisms for medical institutions at all levels, improving medical insurance-related systems, strengthening the promotion of relevant policies of MAs through multiple channels, and guiding patients of all ages to seek medical treatment in a reasonable and orderly manner. As a consequence, the realization of hierarchical diagnosis and treatment might be enhanced.

## Additional file

Additional file1: Questionnaire for patients. The content of questionnaire for patients used in the study. (DOC 41 kb)

## Data Availability

The datasets generated and/or analyzed during the current study are available from the corresponding author on reasonable request. E-mail: mengkai@ccmu.edu.cn.

## Abbreviations

CHC: Community Healthcare Center
NCMS: New rural cooperative medical system
OR: Odds ratio
UEBMI: Urban employees’ basic medical insurance
URBM: Urban residents’ basic medical insurance
