# Supplementary material for: The willingness of patients to make the first visit to primary care institutions and its influencing factors in Beijing medical alliances: a comparative study of Beijing’s medical resource-rich and scarce regions
Source: BMC Health Serv Res. 2019 Jun 7;19:361. doi: 10.1186/s12913-019-4184-0 (PMC6556011; doi:10.1186/s12913-019-4184-0)
Supplement: Supplementary file 1 — Questionnaire for patients. The content of questionnaire for patients used in the study. (DOC 41 kb) [file 12913_2019_4184_MOESM1_ESM.doc]

Hospital Name： Questionnaire Number：

Questionnaire for patients

1. Your gender：A. Male B. Female
2. Your date of birth:
3. Your domicile place:

A. The city's downtown B. suburb of this city C. Not this city

1. Are you an inpatient or an outpatient?
2. Inpatient B. Outpatient
3. The type of medical insurance you participate in

A. Urban Employees Basic Medical Insurance (UEBMI)

B. Urban Residents Basic Medical Insurance (URBMI)

C. National medical insurance (NMI)

D. New rural cooperative medical system (NRCMS)

E. Commercial Insurance (CI)

F. Out of pocket

1. Do you have any chronic diseases confirmed by your doctor?
2. Yes B. No
3. How much is your average monthly real expenditure on health (deducting the personal payment portion after reimbursement)?

A .less than 300 yuan B .301-500 yuan C. 501-800 yuan

D. 801-1000 yuan E. 1001 yuan or more

1. When you think the disease is lighter, what medical institution will you choose?

A. Class-three hospital

B. Class-two hospital

C. Class-one hospital or community health service centre (station)

D. Take some medicine at the pharmacy instead of going to the hospital.

1. When you think the disease is heavy, what medical institution will you choose?

A. Tertiary hospital

B. Secondary hospital

C. Primary hospital or community health service centre (station)

D. Take some medicine at the pharmacy instead of going to the hospital.

1. What do you think are the main problems in medical treatment?(limited to three)

A. Finding a trusted doctor is difficult

B. Long waiting time for seeing a doctor

C. Long time for taking medicine

D. Other hospital beds are difficult

E. Appointment inspection time is long

F. Less time for communication with doctors

G. Complicated treatment process

H. Hospital is far away.

I. Others

1. How much do you know about the related policies of the medical community? (If you choose A, go to item 13)

A. Do not understand very much

B. Not very understanding

C. General understanding

D. More understanding

E. Very much understanding

1. What is your approach to understanding the related policies of the Medical Council or the work plan of the Medical Council?

A. Media reports

B. Community promotion

C. Hospital promotion

D. Relatives and Friends Recommend

E. Other

1. Does the adjustment of the health insurance policy (such as the proportion of reimbursement for medical treatment) affect your medical treatment choice?

A. No effect B. Little effect C. No obvious D. Larger impact E. Large impact

1. Are you willing to make the first visit to primary care institutions?

A. Unwilling B. Relatively unwilling C. Indifferent D. More willing E. Very willing

1. The main reason why you are willing to go to the primary care institutions in the resident area for the first contact care (limited to three items)

A. Convenient, close to home, easy to take care of

B. The proportion of Medicare reimbursement is relatively high, and self-paid expenses are less

C. Treatment environment is more suitable for rehabilitation or follow-up treatment

D. It can be referred to a large hospital through a community hospital

E. Large hospitals can contact community hospitals or rehabilitation hospitals to ensure continuity of treatment

F. Short waiting time at primary care institutions

G. Doctor visits at primary care institutions are more detailed and comprehensive

H. Family doctor-style services can be provided at primary care institutions

I. Four types of chronic diseases enjoy the prescription policy in the community (that is, the amount of drugs that can be opened for two months at a time)

J. Other __________________________

1. The main reason why you are unwilling to go to the primary care institution in the resident area for the first contact care (limited to three items)

A. The process of referral is a waste of time and delay of illness. It is better to go directly to a large hospital.

B. Do not trust the medical conditions and the skills of doctors of the primary care institution

C. The types of drugs are not complete

D. Less inspection inspection items

E. Medicare reimbursement issues (drugs can not be reimbursed, medical service project reimbursement ratio is small)

F. Primary care institution has no beds and cannot provide hospitalization service

G. Other ______________________________

1. Have you received medical alliance services in the resident area?
2. Yes B. No
3. How satisfied are you with these services?

A. Not satisfied B. Less satisfied C. Generally

D. More satisfactory E. Very satisfied

1. What are the conveniences for you after the medical alliance is established? (limited to three items)

A. Did not experience

B. You can make an appointment at a primary care institution to see an expert from hospital

C. Reduced medical expenses paid by yourself

D. Priority for inspections when referring to hospitals through the primary care institution

E. Mutual recognition of inspection results and reduction of duplicate inspections

F. Information sharingbetween medical Institutions

G. Others: ____________________

1. Which services do you think are more attractive in the service projects launched by the Medical alliance (limited to three items)?

A. Experts from large hospitals visit theprimary care institution

B. Experts who can make appointments to large hospitals at the primary care institution

C. In the community can share the inspection equipment in large hospitals, to achieve mutual recognition of inspection results, reduce duplication of inspections

D. Be able to refer to a higher hospital directly in the primary care institution for direct hospitalization, reducing waiting time

E. The green channel forcheck and test (reduce waiting time, exempt from registration fees)

F. The types of drugs in the medical alliance are more abundant, and medicines in large hospitals can also be opened at the primary care institution.

G. Patients in Large hospitals go to the community without recalculating the pay line.

H. It is not necessary to re-enter personal information when referring to a large hospital from a primary care institution, so that the continuity of diagnosis and treatment can be maintained.

I. Other: _____________

1. What kind of convenience do you hope the development of the medical alliance will bring to you in the future? (limited to three items)

A. Experts who can make appointments to large hospitals at the primary care institution

B. Be able to refer to a higher hospital directly in the primary care institution for direct hospitalization, reducing waiting time

C. Mutual recognition of inspection results and reduction of duplicate inspections

D. Increase the variety of drugs in the primary care institution

E. The historical information of the patients can be shared and exchanged in different hospitals within the medical

F. Reduce personal medical expenses

G. Others:_____________

1. What are your opinions and suggestions on the establishment of medical alliances?

______________________________________________________________________________________________________________________________________________________
